# Higher serum haptoglobin levels were associated with improved outcomes of patients with septic shock

**DOI:** 10.1186/s13054-022-04007-y

**Published:** 2022-05-17

**Authors:** Peng Lan, Peihao Yu, Jun Ni, Jiancang Zhou

**Affiliations:** Department of Critical Care Medicine, Sir Run Run Shaw Hospital, Zhejiang University School of Medicine, Hangzhou, China

## Dear Editor,

Regarding the recent study published in *Critical Care* on the role of haptoglobin in acute kidney injury in critically ill adults with ARDS and therapy with VV ECMO [1], we would like to explore the association between serum haptoglobin levels and clinical outcomes of patients in septic shock. Septic shock results in disseminated intravascular coagulation and microvascular perfusion disorders, leading to hemolysis, rendering massive hemoglobin release [2]. Cell-free hemoglobin and its degradation component heme contribute to multiorgan failure and worse clinical outcomes of septic shock [3]. Haptoglobin helps reduce the toxic effects by binding cell-free hemoglobin and the protective role of haptoglobin in sepsis has been confirmed [4]. However, there is no evidence about the role of serum haptoglobin in septic shock. We therefore aimed to explore the association between serum haptoglobin and the prognosis of patients in septic shock.

**Fig. 1:**
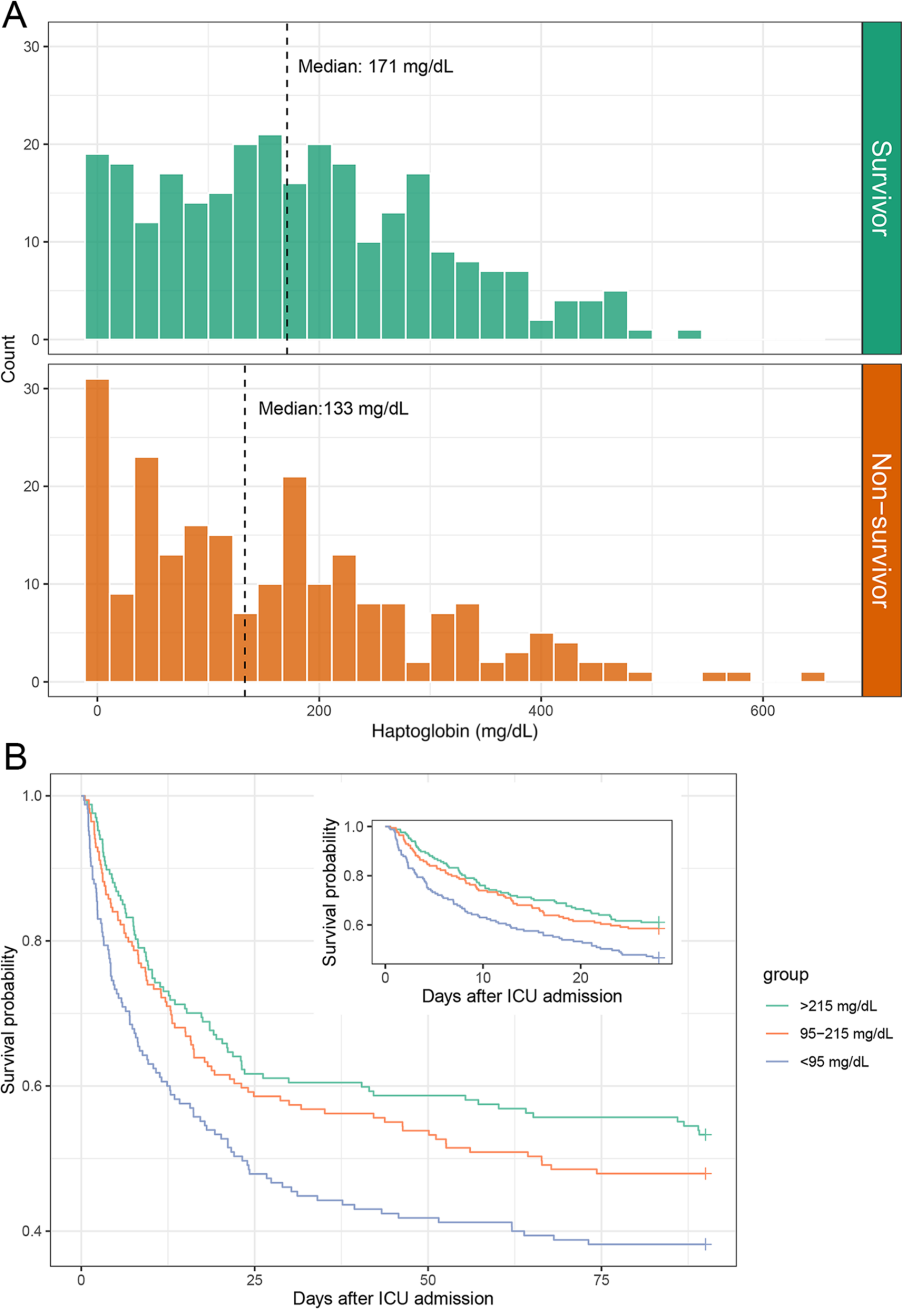
Association between haptoglobin levels and the outcomes of patients with septic shock. **A** Distribution of haptoglobin between survivors and non-survivors based on 28-day mortality. **B** Kaplan–Meier curves showing the association between the haptoglobin levels and the 28-day and 90-day mortality

The Medical Information Mart for Intensive Care III database was employed for analysis. A total of 501 septic shock patients receiving norepinephrine therapy with initial haptoglobin measurements were included (Additional file 1: Figure S1). Age, metastatic cancer, diabetes mellitus, mechanical ventilation and SOFA score were associated with 28-day mortality (Additional file 1: Table S1). The haptoglobin values for 28-day survivors were significantly greater than those for non-survivors (median value: 171 mg/dL vs. 133 mg/dL, *p* = 0.009, Fig. 1A). The haptoglobin level for septic shock patients was significantly greater than those for patients in non-sepsis (median value: 159 mg/dL vs. 124 mg/dL, *p* < 0.001, Additional file 1: Figure S2A). However, with the increasing of severity, the haptoglobin levels significantly decreased for septic shock patients (Additional file 1: Figure S2B, *p* < 0.001 for a trend). All the septic shock patients were categorized into three groups according to the tertiles of haptoglobin values. Higher haptoglobin level was associated with improved 28-day (*p* = 0.018), 90-day (*p* = 0.020), ICU (*p* = 0.002) and hospital (*p* = 0.003) mortality (Additional file 1: Table S2). However, the haptoglobin levels had no impact on the length of stay in ICU and hospital (Additional file 1: Table S2). Survival curve analysis revealed that the higher haptoglobin levels were associated with improved risk of 28-day (Log rank = 10.17, *p* = 0.006) and 90-day (Log rank = 10.7, *p* = 0.005) mortality (Fig. 1B). Haptoglobin levels, age, SOFA score, mechanical ventilation, metastatic cancer and diabetes mellitus were included in a multivariable Cox proportional hazard model to explore the association between haptoglobin levels and 28-day mortality. Compared with the lower tertile of haptoglobin level (< 95 mg/dL), the middle (hazard ratio: 0.737, *p* = 0.051) and upper tertile (hazard ratio: 0.653, *p* = 0.012) of haptoglobin were associated with lower risk of 28-day mortality (Additional file 1: Table S3). Other covariates including older age (hazard ratio: 1.785, *p* < 0.001), SOFA score (hazard ratio: 1.122, *p* < 0.001) and metastatic cancer (hazard ratio: 2.833, *p* < 0.001) were significantly associated with 28-day mortality.

This study confirmed that increased haptoglobin levels were associated with an improved 28-day mortality in septic shock patients receiving norepinephrine therapy. In addition, the haptoglobin level of septic shock patients was significantly higher than those of non-sepsis patients, but it decreased with increasing of illness severity. Due to versatile physiological derangements in septic shock, whether haptoglobin serves as a marker or mediators remains unclear. A previous study has demonstrated that haptoglobin infused significantly improved the animals’ metabolic profile and reduced the severity of shock, as well as risk of death [5]. However, whether haptoglobin supplementation could be a potential therapeutic strategy for septic shock in clinical settings warrants further studies.

## Supplementary Information

**Additional file 1. Table S1:** Baseline characteristics of 501 septic shock patients according to 28-day status. **Table S2:** Clinical outcomes of 501 septic shock patients according to haptoglobin levels. **Table S3:** Cox proportional hazard models exploring the association between haptoglobin and 28-day mortality. **Figure S1:** Flow chart: the inclusion of the study population. A total of 501 septic shock patients receiving norepinephrine therapy with initial haptoglobin measurements were included. **Figure S2:** Haptoglobin comparisons among study populations. (**A**) Initial haptpglobin levels between patients with septic shock and non-sepsis. (**B**) The haptoglobin levels in patient with septic shock against SOFA score categories.

## Data Availability

The data that support the findings of this study are available from MIMIC website.

## Abbreviations

ICU: Intensive care unit
SOFA: Sequential Organ Failure Assessment
IQR: Interquartile range
SD: Standard deviation
ARDS: Acute respiratory distress syndrome
VV-ECMO: Venovenous extracorporeal membrane oxygenation
