# Supplementary material for: Higher serum haptoglobin levels were associated with improved outcomes of patients with septic shock
Source: Crit Care. 2022 May 17;26:131. doi: 10.1186/s13054-022-04007-y (PMC9112476; doi:10.1186/s13054-022-04007-y)
Supplement: Supplementary file 1 — Additional file 1. Table S1: Baseline characteristics of 501 septic shock patients according to 28-day status. Table S2: Clinical outcomes of 501 septic shock patients according to haptoglobin levels. Table S3: Cox proportional hazard models exploring the association between haptoglobin and 28-day mortality. Figure S1: Flow chart: the inclusion of the study population. A total of 501 septic shock patients receiving norepinephrine therapy with initial haptoglobin measurements were included. Figure S2: Haptoglobin comparisons among study populations. (A) Initial haptpglobin levels between patients with septic shock and non-sepsis. (B) The haptoglobin levels in patient with septic shock against SOFA score categories. [file 13054_2022_4007_MOESM1_ESM.docx]

| Table S1 Baseline characteristics of 501 septic shock patients according to 28-day status | | | | |
| --- | --- | --- | --- | --- |
| Characteristics | Total (n=501) | Survivor (n=278) | Non-survivor (n=223) | p-value |
| Gender (Male, n, %) | 267 (53.3) | 144 (51.8) | 123 (55.2) | 0.510 |
| Age (>=60 yr, n, %) | 302 (60.3) | 148 (53.2) | 154 (69.1) | <0.001 |
| Admission type (n, %) |  |  |  | 0.438 |
| Elective | 11 (2.2) | 8 (2.9) | 3 (1.3) |  |
| Emergency | 476 (95.0) | 261 (93.9) | 215 (96.4) |  |
| Urgent | 14 (2.8) | 9 (3.2) | 5 (2.2) |  |
| Comorbidity (n, %) |  |  |  |  |
| Chronic pulmonary diseases | 93 (18.6) | 44 (15.8) | 49 (22.0) | 0.100 |
| Metastatic cancer | 29 (5.8) | 10 (3.6) | 19 (8.5) | 0.031 |
| Renal failure | 101 (20.2) | 54 (19.4) | 47 (21.1) | 0.729 |
| Diabetes mellitus | 145 (28.9) | 70 (25.2) | 75 (33.6) | 0.048 |
| Hypertension | 79 (15.8) | 45 (16.2) | 34 (15.2) | 0.870 |
| Congestive heart failure | 178 (35.5) | 94 (33.8) | 84 (37.8) | 0.423 |
| Support therapies on admission (n, %) |  |  |  |  |
| Mechanical Ventilation | 336 (67.1) | 171 (61.5) | 165 (74.0) | 0.004 |
| Renal replacement therapy | 45 (9.0) | 24 (8.6) | 21 (9.4) | 0.883 |
| SOFA score (median, IQR) | 9 (7-12) | 8 (6-11) | 11 (8-14) | <0.001 |
| Abbreviations: SOFA: sequential organ failure assessment; IQR: Interquartile range | | | | |

| Table S2 Clinical outcomes of 501 septic shock patients according to haptoglobin levels | | | | | |
| --- | --- | --- | --- | --- | --- |
| Outcomes | Total (n=501) | Haptoglobin  (<95 mg/dL, n=165) | Haptoglobin  (95-215 mg/dL, n=169) | Haptoglobin  (>215 mg/dL, n=167) | p-value |
| Mortality (n, %) | | | | | |
| 28-day | 223 (44.5) | 88 (53.3) | 70 (41.4) | 65 (38.9) | 0.018 |
| 90-day | 268 (53.5) | 102 (61.8) | 88 (52.1) | 78 (46.7) | 0.020 |
| ICU | 199 (39.7) | 84 (50.9) | 57 (33.7) | 58 (34.7) | 0.002 |
| Hospital | 228 (45.5) | 93 (56.4) | 68 (40.2) | 67 (40.1) | 0.003 |
| Length of stay (median days, IQR) | | | | | |
| ICU | 6.1 (2.9-12.7) | 5.48 (2.56-13.4) | 6.10 (2.87-11.8) | 6.13 (3.04-13.9) | 0.481 |
| Hospital | 10.8 (6.0-19.5) | 10.4 (4.86-21.6) | 10.7 (6.80-17.3) | 11.0 (6.03-19.9) | 0.657 |
| Abbreviations: ICU: Intensive care unit; IQR: Interquartile range | | | | | |

| Table S3 Cox proportional hazard models exploring the association between haptoglobin and 28-day mortality. | | | |
| --- | --- | --- | --- |
| Factors | Hazard ratio | 95% CI | p-value |
| Haptoglobin |  |  |  |
| <95 mg/dL | Reference | Reference | Reference |
| 95-215 mg/dL | 0.737 | 0.527-1.001 | 0.051 |
| >215 mg/dL | 0.653 | 0.468-0.910 | 0.012 |
| Age >60 y | 1.785 | 1.337-2.384 | <0.001 |
| SOFA score | 1.122 | 1.083-1.163 | <0.001 |
| Mechanical Ventilation | 1.207 | 0.879-1.656 | 0.245 |
| Metastatic cancer | 2.833 | 1.739-4.614 | <0.001 |
| Diabetes mellitus | 1.226 | 0.926-1.622 | 0.154 |
| Abbreviations: SOFA: sequential organ failure assessment; CI: Confidence interval | | | |





Figure S1. Flow chart: the inclusion of the study population. A total of 501 septic shock patients receiving norepinephrine therapy with initial haptoglobin measurements were included.


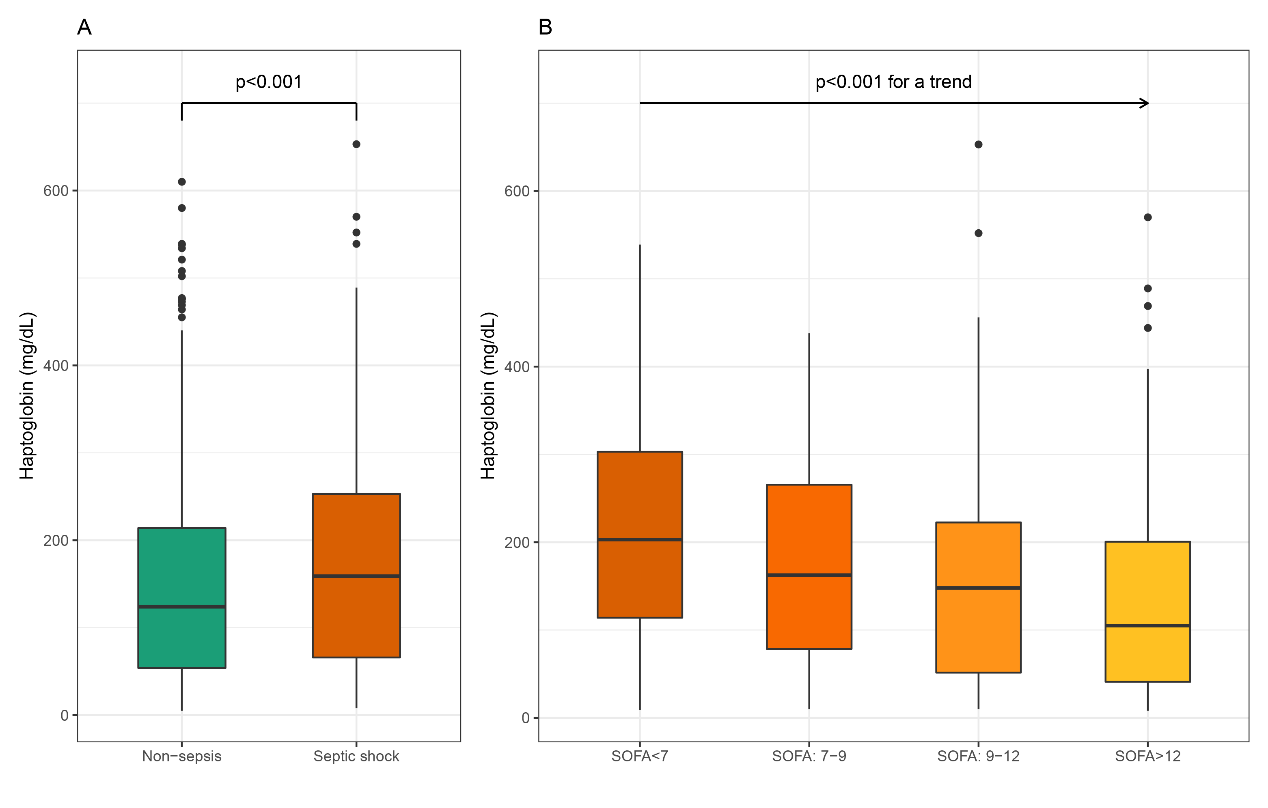


Figure S2. Haptoglobin comparisons among study populations. (A) Initial haptpglobin levels between patients with septic shock and non-sepsis. (B) The haptoglobin levels in patient with septic shock against SOFA score categories.
